# College Matters, Telescope, N. W. Review

**Published:** 1858

**Authors:** 


### Telescope.

The Trustees and Faculty of the Medical Department of the Iowa University, would acknowledge the receipt of a fine Telescope presented to the Institution by Mr. Saville, formerly of Ind., now of Nebraska Ter'y. This fine instrument—with one of Azoo's fine dissecting Manakin's life size—received since the opening of the session, will add much to our collection.

